# ADRD: Detecting diffusion-generated images via adversarial perturbation induced reconstruction discrepancy

**DOI:** 10.1371/journal.pone.0350655

**Published:** 2026-07-14

**Authors:** Yi Zhou, Xiangwei Hu, Jun Tong, Changting Lin, Mei Sun, Shuangxi Chen

**Affiliations:** 1 School of Internet, Jiaxing Vocational and Technical College, Jiaxing, Zhejiang, China; 2 Jiaxing Municipal Public Security Bureau, Jiaxing, Zhejiang, China; 3 Binjiang Institute of Zhejiang University, Hangzhou, Zhejiang, China; Khalifa University, UNITED ARAB EMIRATES

## Abstract

The rapid advancement of diffusion models has raised concerns about their misuse in generating deceptive visual content, motivating growing interest in AI-generated image detection. Many existing detection methods rely on image semantic features, but modern diffusion models are optimized to closely match the semantic structure of real images, reducing the effectiveness of semantic-based detection. An alternative line of work exploits differences revealed through diffusion reconstruction; however, most existing approaches treat reconstruction error as a static and passive metric, which can be sensitive to generators or post-processing, thereby limiting robustness. In this work, we propose Adversarial Diffusion Reconstruction Distance (ADRD), a detection framework that models diffusion reconstruction as a dynamic response process rather than a fixed descriptor. ADRD actively probes the reconstruction behavior by introducing perturbation in latent space and measuring how reconstruction deviations respond under identical perturbations. We empirically observe that real images typically exhibit larger and more variable reconstruction responses, while diffusion-generated images tend to show more stable reconstruction behavior, reflecting differences in their alignment with the diffusion model’s implicit data manifold. By characterizing reconstruction sensitivity instead of absolute reconstruction error, ADRD provides a complementary perspective to existing reconstruction-based detectors. Experimental evaluations on multiple benchmarks suggest that reconstruction response under controlled perturbations constitutes a meaningful signal for diffusion-generated image detection. The code is available at https://github.com/ezell-chou/adrd

## Introduction

The advent of AI-generated image technology has revolutionized visual content synthesis through deep learning models that approximate real image distributions. Following the groundbreaking development of diffusion models [1], generative models such as Midjourney [2] and the DALL-E series [3,4] have achieved remarkable advancements in both image fidelity and generation controllability. Their outputs now approach perceptual indistinguishability from authentic images by human observers. While this technological leap has spurred innovation in artistic and design domains, it has significantly lowered the technical barrier to producing malicious content.

The abuse of synthesis image technology has given rise to a range of multi-dimensional security threats: 1) **Disruption of the information ecosystem:** The dissemination of forgeries has demonstrably impacted real-world systems. For instance, a diffusion-generated image depicting an explosion at the Pentagon caused significant turmoil in global financial markets [5]. 2) **Privacy violations via synthetic identities:** The malicious application of DeepFake technology, particularly exemplified by the non-consensual generation and dissemination of explicit imagery in South Korea, has inflicted psychological harm while testing legal and ethical frameworks [6,7]. 3) **Copyright infringement risks:** Style-transferred images increasingly challenge intellectual property protections, threatening artistic innovation ecosystems. 4) **Unregulated harmful content:** Unrestricted generation risks amplifying violent, extremist, and illegal visual materials. These threats collectively erode digital content trustworthiness and precipitate structural crises in social trust architectures.

Against this backdrop, reliable detection of AI-generated images has become critical for content security. This paper addresses the task of: given an input image *x* of unknown origin, determining whether it originates from the real image distribution $p_{r}(x)$ or a generative model distribution $p_{g}(x)$ without access to generation metadata. Unlike detection methods targeting single GAN architectures, real-world applications typically encounter more complex scenarios including cross-generator generalization, distribution shifts, and common post-processing/degradation—all of which degrade detectable statistical traces and compromise detection performance.

Current detection methodologies face evolving challenges. Prior to diffusion models, approaches primarily targeted GAN-generated images [8–12], proving inadequate against modern diffusion model outputs. Since 2023, research has been bifurcated into 2 primary perspectives: Some studies start from investigations of frequency-domain anomalies [13–16] and model-specific texture patterns [17–19] in AI-generated images. Another leverages vision-language models like CLIP [20] to enhance generalization through cross-modal alignment analysis [21–24]. While these methods provide valuable solutions to the rapidly evolving generative landscape, detection signal stability remains vulnerable in practical deployment. Thus, exploring signals complementary to existing traces—verifiable under unified protocols—retains significant relevance.

**Fig 1:**
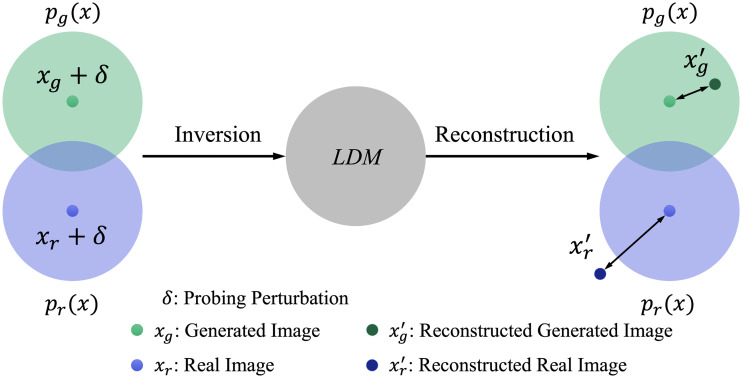
Adversarial diffusion reconstruction distance.

We propose the **A**dversarial **D**iffusion **R**econstruction **D**istance (ADRD), a novel detection framework for distinguishing between authentic and diffusion-generated images. Our approach originates from an empirical observation: the inversion-reconstruction process of diffusion models acts as a prior-constrained mapping that “pulls” inputs toward the statistical regularities learned by the model. Consequently, reconstruction exhibits differential sensitivity to input perturbations depending on whether the input originates from real or generated distributions. Based on this insight, we introduce an active latent-space probing perturbation. Under identical conditions, we compare input images with their diffusion-reconstructed counterparts to derive detection signals, as shown in Fig 1. Conceptually, this design explicitly quantifies sensitivity to controlled perturbation during reconstruction, providing complementary discriminative evidence.

Given real image distribution $p_{r}\left(x\right)$ and diffusion-generated image distribution $p_{g}\left(x\right)$, we sample $x_{r}$ and $x_{g}$ respectively. After applying an identical probing perturbation, the input images undergo diffusion inversion and reconstruction in latent space, yielding reconstructed outputs $\overset{\prime }{\underset{r}{x}}$ and $\overset{\prime }{\underset{g}{x}}$. This process characterizes differential inversion responses to controlled perturbation across input distributions, where the reconstruction error reflects stability differences in the diffusion model’s response to input variations.

Our methodology unfolds in 3 stages: 1) Optimized Probe Generation: Learn a controlled probing perturbation in diffusion model’s latent space via optimization to maximize reconstruction error between real and diffusion-generated images. 2) Perturbed Reconstruction: Apply the probe to VAE-encoded latent representations, then perform diffusion inversion and reconstruction. 3) Difference Classification: Train a lightweight classifier on the reconstruction discrepancy to effectively separate real and diffusion-generated images.

This paper proposes ADRD, a diffusion-generated image detection method that constructs discriminative features from the differences of diffusion reconstruction under active probing perturbation. Evaluated on datasets including GenImage and WildFake, experimental results show that ADRD learns a latent-space perturbation under standard training protocols, and exhibits moderate transferability across different generators. At the same time, it shows performance variations under unseen generators or strong degradations. Overall, ADRD achieves detection performance comparable to representative existing methods. More importantly, it offers a complementary perspective to approaches based on static reconstruction error or semantic-level correlations. By quantifying reconstruction sensitivity via active probing perturbation, it provides a reference framework for enhancing diffusion reconstruction-based detection.

Our contributions are summarized in the following aspects:

1. Novel Detection Framework: ADRD introduces optimized latent-space perturbation and uses reconstruction error under perturbation as its core signal, enhancing discriminability under unified reconstruction settings—unlike methods operating directly in pixel or latent spaces.
2. Comprehensive Empirical Evaluation: We report ADRD’s cross-generator performance on GenImage/WildFake under unified protocols. Comparative results with multiple baselines show ADRD matches existing methods on several generators, though effectiveness varies with training generator selection and distribution shifts.
3. Boundary Analysis via Ablation Studies: We examine impacts of probe design, training sample size, reconstruction strength, and classifier architecture. Evaluations under JPEG compression and Gaussian blur clarify ADRD’s operational boundaries, providing empirical guidance for future improvements and ensemble detection.

## Related work

### Diffusion models and inversion mechanisms

The evolution of generative visual models has witnessed diffusion models establish dominance through theoretical innovation and engineering breakthroughs. While pioneering Generative Adversarial Networks (GANs) [8] achieved breakthroughs in image quality, their inherent training instability and mode collapse limitations motivated the exploration of alternative paradigms. The seminal work by Ho et al. [25] on Denoising Diffusion Probabilistic Models (DDPMs) marked a technological inflection point, delivering high-fidelity image synthesis with unprecedented training stability.

Subsequent research has turned attention to diffusion models to refine architecture [1,26], accelerate sampling [27,28], and explore downstream tasks [29,30]. Song et al. [31] pioneered Denoising Diffusion Implicit Models (DDIMs), introducing non-Markovian sampling processes that simultaneously enhance sampling speed and output quality. Dhariwal et al. [1] systematically optimized model architectures with extensive experiments, achieving superior performance via classifier-guided sampling. Rombach et al. [26] later proposed Latent Diffusion Models (LDMs), a pivotal development that maintains image quality while substantially reducing computational requirements.

Recent advancements in diffusion models extend to conditional generative models, particularly text-to-image synthesis. Models like DALL-E [3,4] and Imagen [32] leverage the inherent stability of diffusion processes to align visual outputs with textual descriptions via progressive denoising.

Now, the diffusion model has formed a complete technical system encompassing basic theories [25,31], computational optimizations [1,26–28], and multimodal applications [3,4,32]. Compared to GANs [8–12], diffusion models exhibit significant advantages in terms of training stability and generative controllability. More importantly, their iterative noise-adding and denoising structure naturally supports inversion and reconstruction processes, enabling input images to be mapped into a latent noise space and reconstructed. Although these inversion mechanisms were initially primarily used for image editing and generative control, recent research shows that they have become essential tools for analyzing and detecting diffusion-generated images.

### Detection of diffusion-generated images

The fundamental challenge in AI-generated image detection lies in distinguishing whether an image is sourced from the physical world or algorithmically synthesized content. Early methods primarily targeted GAN-generated images, relying on frequency domain artifacts [13–16] or local texture inconsistencies [17–19]. However, the rapid proliferation of diffusion models has significantly diminished the effectiveness of these GAN-specific detectors.

For diffusion-generated images, recent research has progressively introduced multimodal semantic understanding and feature fusion strategies. Approaches such as CLIP-driven feature alignment [20,21], multi-stream fusion architectures [22,23], and the construction of adversarial training sets with reverse image search [24] have enabled precise identification of cross-modal semantic contradictions. Meanwhile, dynamic texture features integrated with semantic contexts [33] and multi-scale semantic pyramids architectures [34] have further enhanced the ability to detect subtle forgery traces.

Additionally, reconstruction-based detection has emerged as a dedicated approach specifically designed for diffusion models. DIRE [35] pioneered the use of pixel-level reconstruction errors from diffusion inversion to reveal the instability of generated images. DNF [36] and LaRE^2^ [37] improved detection efficiency and performance by leveraging inverse diffusion noise statistics and latent space reconstruction errors, respectively. FakeInversion [24] demonstrated that inversion instability is more pronounced for images generated by unseen text-to-image models. FIRE [38] further enhanced detection discriminability by analyzing reconstruction errors through frequency decomposition. Although existing methods have validated the effectiveness of reconstruction discrepancies, most treat reconstruction errors as static features and have yet to systematically analyze reconstruction stability under controlled input variations.

### Stability and sensitivity of diffusion reconstructions

The stability issues in the inversion and reconstruction processes of diffusion models have garnered increasing attention in recent years. Unlike traditional discriminative models, the reverse diffusion process typically involves multi-step numerical approximations, rendering its trajectory potentially sensitive to initial conditions and data distributions. Liu et al. [28] provided foundational theoretical analysis by examining the numerical properties of diffusion models on data manifolds, revealing that the reverse diffusion process is notably sensitive to initial perturbations and approximation errors.

At the application level, inconsistent behaviors induced by input distribution discrepancies have been observed in diffusion inversion and image editing tasks. Parmar et al. [29] discovered significant variations in how different input images respond to editing operations after diffusion inversion, indicating high sensitivity to distribution shifts. Furthermore, Salman et al. [39] demonstrated that even minor input perturbations can be progressively amplified during diffusion generation and editing, substantially affecting final outcomes.

Related research indirectly corroborates this phenomenon from perspectives of data protection and generative control. Multiple studies have shown that introducing structured perturbations or protective noise at the input stage can significantly alter inversion trajectories and generation results, confirming that diffusion models lack robustness in reconstruction consistency when facing input variations [40–43].

In summary, existing work has revealed the sensitivity of diffusion model inversion to input variations and distribution shifts through theoretical analysis and perturbation experiments. However, this stability discrepancy has yet to be systematically modeled as a discriminative signal for detecting generated images.

### Proposed method

In this section, we describe the proposed ADRD framework. Our method is based on the modeling assumption that real images and diffusion-generated images exhibit systematically different reconstruction behaviors under the same diffusion reconstruction operator. The diffusion reconstruction process can be viewed as an approximate projection onto the image manifold implicitly learned by the diffusion model. While diffusion-generated images naturally conform to this manifold, real images may deviate from it due to restricted model capacity and distribution shifts.

To expose this difference, we introduce a controlled perturbation in the latent space as a probe, which is applied uniformly to both real and diffusion-generated images. The probing perturbation is not intended to alter image semantics, but to amplify the intrinsic discrepancy in reconstruction stability between the two image sources. We then quantify this discrepancy by measuring the difference between the original images with probe and their reconstructed counterparts with probe, forming the ADRD, which serves as the detection criterion.

### Problem formulation

Given a diffusion model parameterized by *θ*, its reconstruction process can be formulated as the reconstruction operator $D_{\theta }\left(L(x)\right)$. Here, x denotes the input image, L(·) represents the forward noising process, and $D_{\theta }(\cdot )$ is the reverse denoising process. This operator can be viewed as an approximate projection that maps input samples onto the image manifold implicitly learned by the diffusion model. Since diffusion-generated images inherently conform to this manifold structure while real images typically reside outside or near its boundary, the two categories exhibit systematic differences in diffusion reconstruction stability.

**Fig 2:**
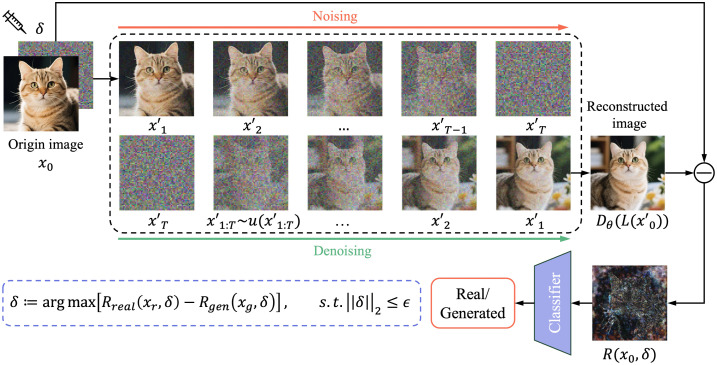
Detection process for diffusion-generated images.


To amplify this divergence, we introduce an active probing perturbation δ in the latent space and uniformly apply it to the latent representations of real images $x_{r}$ and diffusion-generated images $x_{g}$ as shown in Fig 2. Based on the diffusion reconstruction results, we define their respective reconstruction errors as:

$$\begin{array}{c} R_{real}(\delta )={\left\|x_{r}+\delta -R_{\theta }\left(x_{r}+\delta \right)\right\|}_{\text{2}} \end{array}$$

$$\begin{array}{c} R_{gen}(\delta )={\left\|x_{g}+\delta -R_{\theta }\left(x_{g}+\delta \right)\right\|}_{\text{2}} \end{array}$$

The objective of this work is to learn an optimal latent space probing perturbation δ that maximizes the difference in reconstruction errors between real and diffusion-generated images under identical perturbation conditions:


$$\begin{array}{c} \delta :=\text{arg}\text{max}\left[R_{real}\left(x_{r},\delta \right)-R_{gen}\left(x_{g},\delta \right)\right],\;s.t.\;{\left||\delta |\right|}_{\text{2}}\leq \epsilon \end{array}$$

where ϵ constrains the perturbation magnitude to avoid introducing perceptible changes.

An active probing perturbation *δ* is superimposed onto the original image. A Latent Diffusion Model (LDM) is then employed as a pre-trained diffusion model to reconstruct the original image in latent space. The reconstruction error is computed as the difference between original image and its reconstruction. The optimized perturbation *δ* is obtained through iterative optimization algorithms, with the objective of maximizing the difference in reconstruction errors between real and diffusion-generated images.

It should be noted that the reconstruction operator $D_{\theta }\left(L(x)\right)$ involved in the optimization objective above is inherently a stochastic process. Due to the random noise introduced during the forward noising and reverse denoising processes, the output of diffusion reconstruction varies across different noise realizations. Consequently, the reconstruction errors $R_{real}(\delta )$ and $R_{gen}(\delta )$ can be regarded as random variables due to stochastic diffusion trajectories. The difference term corresponds to an estimation of the expected reconstruction error under the distribution of stochastic diffusion trajectories. This stochasticity makes it challenging to obtain the gradient of the objective function with respect to the probing perturbation δ in an analytical form.

Therefore, this work adopts a Monte Carlo strategy to approximate the gradient estimation. By sampling *N* independent diffusion trajectories, the gradient is estimated as:


$$\begin{array}{c} \nabla_{\delta }E_{\overset{\prime }{\underset{\text{1}:T}{x}}\sim u\left(\overset{\prime }{\underset{\text{1}:T}{x}}\right)}[R_{real}(\delta )-R_{gen}(\delta )]\approx \frac{\text{1}}{N}\sum_{i=\text{1}}^{N}\nabla_{\delta }[\overset{\left(i\right)}{\underset{real}{R}}(\delta )-\overset{\left(i\right)}{\underset{gen}{R}}(\delta )] \end{array}$$

where $\overset{\prime }{\underset{\text{1}:T}{x}}$ represents the latent variable representations during the denoising process, and $u\left(\overset{\prime }{\underset{\text{1}:T}{x}}\right)$ denotes the distribution of $\overset{\prime }{\underset{\text{1}:T}{x}}$.

This approach renders the optimization problem practically solvable, ensuring computational feasibility while providing stable gradient estimates for subsequent perturbation optimization.

After approximating the gradient of the reconstruction error difference, this work employs a constrained iterative optimization strategy to update the latent space probing perturbation δ. Specifically, we utilize projected gradient ascent method, where at each iteration the perturbation is updated along the direction that amplifies the reconstruction difference between real and diffusion-generated images. This update strategy draws inspiration from the FGSM [44] but is adapted to the maximization objective of this work. The iterative update rule is expressed as:


$$\begin{array}{c} \delta^{\left(t+\text{1}\right)}=\Pi_{\epsilon }\left\{\delta^{\left(t\right)}+\alpha \cdot \text{sgn}\left[\nabla_{\delta }\left(R_{real}\left(x_{r},\delta \right)-R_{gen}\left(x_{g},\delta \right)\right)\right]\right\} \end{array}$$

where α is the step size parameter, sign(·) is the sign function (providing stable gradient direction), and $\Pi_{\varepsilon }(\cdot )$ denotes the projection operator that constrains the perturbation within an 2-norm ball of radius ϵ.

This iterative optimization is performed in latent space, ensuring relatively stable convergence behavior even in the presence of noisy stochastic gradient estimates. Through multiple iterative updates, an optimal latent-space probing perturbation δ that maximizes the reconstruction error difference under constraints is progressively obtained. This serves as a critical discriminative signal for subsequent reconstruction-difference-based detection methods.

This process is summarized in Algorithm 1. By sampling different Gaussian noises in latent space and performing gradient ascent on the computed image reconstruction error across multiple samples, the maximized image difference value is achieved.

**Algorithm 1. Probing perturbation optimization**

**Input:** Real image $x_{r}$, Diffusion-generated image $x_{g}$, Diffusion model parameters θ,

Number of Monte Carlo N, Step Length α, **Perturbation budget** ϵ, **Number of iterations** ***T***

**Output:** Optimized probing perturbation δ*

**Initialize** $\delta^{\left(0\right)}$ **← 0**

**for *t* = 0 to *T* – 1 do**

   **grad ← 0**

   **for** ***i*** **= 1 to** ***N*** **do**

      $\overset{\left(i\right)}{\underset{r,1:T}{x}}\sim q\left(x_{1:T}|x_{r}+\delta^{\left(t\right)}\right)$, $\overset{\left(i\right)}{\underset{g,1:T}{x}}\sim q\left(x_{1:T}|x_{g}+\delta^{\left(t\right)}\right)$

      $\overset{\left(i\right)}{\underset{real}{R}}\leftarrow {\left|\left|x_{r}+\delta^{\left(t\right)}-\overset{\left(i\right)}{\underset{r,recon}{x}}\right|\right|}_{2}$

      $\overset{\left(i\right)}{\underset{gen}{R}}\leftarrow {\left|\left|x_{g}+\delta^{\left(t\right)}-\overset{\left(i\right)}{\underset{g,recon}{x}}\right|\right|}_{2}$

      $grad\leftarrow grad+\nabla_{\delta }(\overset{\left(i\right)}{\underset{real}{R}}-\overset{\left(i\right)}{\underset{gen}{R}})$

   **end for**

   $\begin{array}{c} grad\;\leftarrow \;grad\;/\;N \end{array}$

   $\delta^{\left(t+1\right)}\leftarrow \delta^{\left(t\right)}+\alpha \cdot sign\left(grad\right)$

   $\begin{array}{c} \delta^{\left(t+1\right)}\leftarrow \delta^{\left(t\right)}\cdot min\left(1,\epsilon /{\left|\left|\delta^{\left(t\right)}\right|\right|}_{2}\right) \end{array}$

**end for**

**return** $\delta^{*}\leftarrow \delta^{\left(T\right)}$

### Universal latent-space probing perturbation

In Section 3.1, we discussed a method for learning latent-space probing perturbation tailored to a single pair of real and diffusion-generated images. However, in practical diffusion-generated image recognition scenarios, we do not have prior knowledge of which diffusion model was used to generate a given image. Therefore, probing perturbation learned for a single image pair or a specific generative model may lack generalization capability in real-world applications.

To address this, we propose a strategy for learning a universal latent-space probing perturbation. The core idea is to jointly model the reconstruction difference behavior across multiple pairs of real and diffusion-generated images during perturbation optimization, directly learning a unified perturbation that remains effective under diverse samples and multiple models.

Specifically, let $\left\{\overset{N}{\underset{i=\text{1}}{\left(\overset{\left(i\right)}{\underset{r}{x}},\overset{\left(i\right)}{\underset{g}{x}}\right)}}\right\}$ denote a collection of image pairs comprising real images and images generated by different diffusion models. We introduce a shared probing perturbation δ in latent space and apply it simultaneously to the latent representations of all samples. Based on the diffusion reconstruction results, we define the difference in reconstruction errors between real and generated images and optimize the expected value of this difference over the sample set.

**Fig 3:**
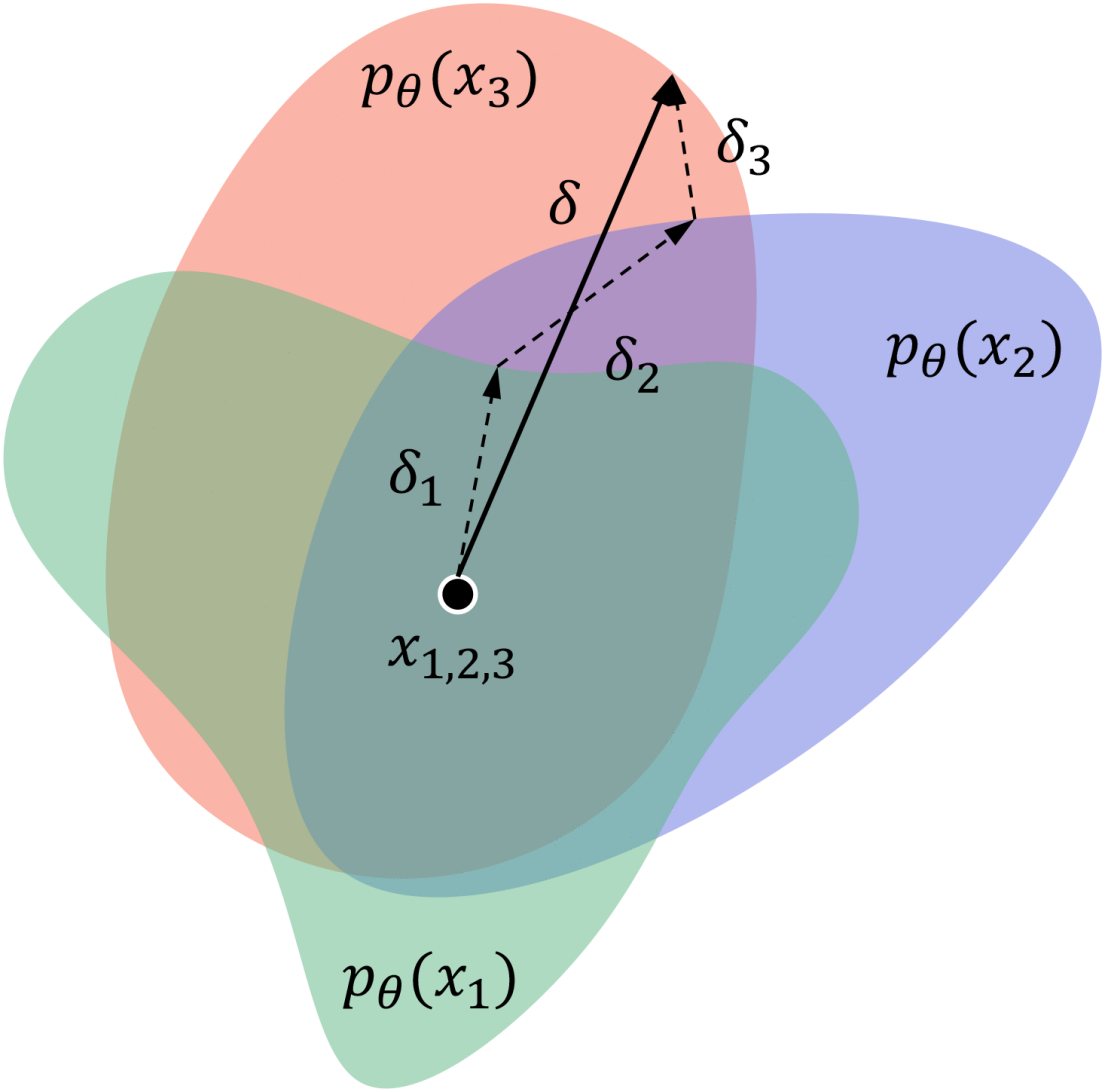
Schematic diagram for calculating the universal probing perturbation.

As illustrated in Fig 3, this universal probing perturbation can be interpreted as seeking a direction in latent space where the reconstruction response of diffusion-generated images remains relatively stable, while the reconstruction deviation of real images is significantly amplified.

Color-coded regions represent the latent-space distributional differences $p_{\theta }(x_{i})$ between real images and different diffusion-generated images after diffusion reconstruction. Given multiple pairs of real and diffusion-generated images, a shared probing perturbation δ is introduced in latent space and uniformly applied to all sample images. Through joint optimization, probing perturbation systematically amplifies the differences between real and generated images during diffusion reconstruction. The dashed arrows indicate the optimized probe direction (maximizing the difference) within the latent-space distributional differences of different diffusion-generated and real image pairs.

In practical optimization process, we directly adopt the Monte Carlo gradient estimation and projected gradient ascent strategy described in Algorithm 1. The key adaptation is that during each gradient update, we average the reconstruction error gradients across multiple sample pairs to jointly optimize every shared probing perturbation.

### Diffusion reconstruction and difference metric

**Fig 4:**
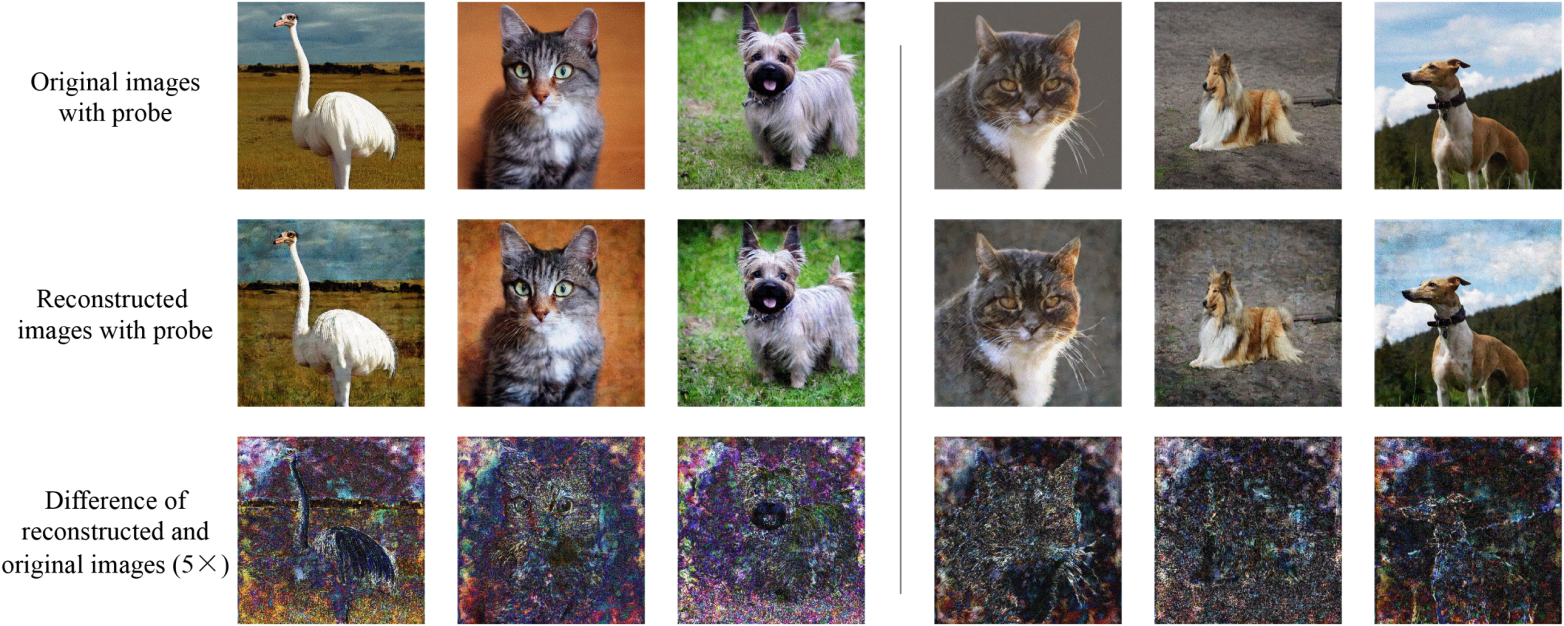
Comparison of ADRD differences between real and diffusion-generated images. **(A)** Difference after real image reconstruction. **(B)** Difference after generated image reconstruction.

We observe that the reconstruction operator of diffusion models exhibits distinct stability characteristics for real versus diffusion-generated images. Specifically, real images show significantly greater displacement from their original inputs after reconstruction compared to diffusion-generated images. To actively amplify this discrepancy, we introduce a probing perturbation in latent space. As demonstrated in Fig 4, measurable differences emerge in the error between reconstructed images and originals. Diffusion-generated images exhibit a higher proportion of black pixels upon reconstruction, suggesting smaller pixel-space differences compared to real images.

Convert reconstructed images to pixel space and compute the absolute difference (magnified 5×). Observations reveal that diffusion-generated images (right) exhibit a higher proportion of black pixels compared to real images (left) under identical probing perturbation. This visually evident contrast indicates distinct stability behavior of the diffusion reconstruction operator for genuine versus generated inputs.

The specific method is as follows: Given an input image $x_{0}$, we first map it to latent space via a VAE module and apply the learned universal probe δ, yielding the perturbed representation $\overset{\prime }{\underset{\text{0}}{x}}=x_{\text{0}}+\delta$. We then execute a full diffusion reconstruction process with pretrained LDM without any fine-tuning. As illustrated in Fig 2, The diffusion process progressively corrupts $\overset{\prime }{\underset{0}{x}}$ through T-step Gaussian noise injection, ultimately yielding isotropic Gaussian-distributed noise image $\overset{\prime }{\underset{T}{x}}$. Subsequent reconstruction via the denoising process recovers the image as $D_{\theta }\left(I\left(\overset{\prime }{\underset{0}{x}}\right)\right)$. By comparing the differences between $\overset{\prime }{\underset{0}{x}}$ and $D_{\theta }\left(I\left(\overset{\prime }{\underset{0}{x}}\right)\right)$, real images and diffusion-generated images can be effectively distinguished.

Thus, we present the definition of ADRD:


$$\begin{array}{c} ADRD\left(x_{0}\right)=\left\|\overset{\prime }{\underset{0}{x}}-D_{\theta }\left(I\left(\overset{\prime }{\underset{0}{x}}\right)\right)\right\| \end{array}$$

$$\begin{array}{c} where\;\overset{\prime }{\underset{0}{x}}=x_{0}+\delta \end{array}$$

Where I(·) represents the sampling process in diffusion reconstruction, and $D_{\theta }(\cdot )$ represents the denoising process in diffusion reconstruction. This metric stably captures differences in geometric relationships between input images and the diffusion model manifold.

### Neural network classifier

After completing diffusion reconstruction and computing the differences between input images and their reconstructed outputs, we treat the resulting difference tensor as a structured response map that characterizes reconstruction stability relative to the diffusion model manifold. To transform this difference map into a discriminative decision signal, we introduce a lightweight convolutional neural network as a binary classifier to distinguish real images from diffusion-generated ones.

The classifier employs a multi-layer convolutional architecture to hierarchically extract local and global statistical features from the difference tensor. It is trained with a simple binary cross-entropy loss:


$$\begin{array}{c} \text{L}\left(y,y^{\prime }\right)=-\sum_{i=\text{1}}^{N}\left(y_{i}log\left(\overset{\prime }{\underset{i}{y}}\right)+(\text{1}-y_{i})log\left(\text{1}-\overset{\prime }{\underset{i}{y}}\right)\right) \end{array}$$

where *N* is the batch size, y denotes ground truth labels, and $y^{\prime }$ represents the classifier’s predictions.

Critically, this classifier serves solely as a discriminator for difference representations. Its purpose is to evaluate the separability of features derived from ADRD, not to rely on complex model architectures for detection.

### Experiments

This section presents the experimental configuration and evaluation protocol used to assess the proposed detection method. We conduct a series of experiments across multiple benchmark datasets and generation settings to examine the effectiveness, generalization, and robustness of the approach under consistent training and testing conditions.

### Experimental configuration

#### Datasets.

The experimental data are derived from GenImage [45] and WildFake [46] datasets. GenImage comprises 8 generator-specific subsets (ADM, BigGAN, Glide, Midjourney, SD v1.4, SD v1.5, VQDM, Wukong), each containing an equal number of generated and real images sourced from ImageNet. WildFake includes 8 diffusion-generated subsets (ADM, DALL·E, DDIM, DDPM, Imagen, Midjourney, SD, VQDM). Real images in WildFake originate from multiple open-source datasets, such as Laion-5B, COCO, ImageNet.

In training stage, we follow a single-generator training protocol. For each experiment, one generator subset is exclusively selected. From the subset, 500 diffusion-generated images and 500 real images (1,000 total) are randomly sampled, which are split into training(70%) and validation(30%) sets.

#### Baselines.

1) CNNSpot (CVPR’2020) [47] proposed a CNN generated image detection based on ResNet-50 classifier, which trained on a CNN dataset and generalized to other synthetic image datasets. 2) GramNet (CVPR’2020) [48] employs Gram matrix features to represent the texture and style of images, and combines Gram matrix features at different levels in ResNet structure to improve the detector’s generalization. 3) UnivFD (CVPR’2023) [49] proposed a synthetic image detection based on the feature space of a pre-trained model with the nearest neighbor and linear probing methods. 4) LNP (ECCV’2022) [16] extracts the noise pattern of spatial images based on a trained denoising framework. Then, it distinguishes the AI-generated image from the frequency domain of the noise pattern. 5) DIRE (ICCV’2023) [35] proposed an image representation method named Diffusion Reconstruction Error, to discriminate the diffusion-generated and real images. 6) AIDE (ICLR’2025) [50] proposed a diffusion-generated image detector based on CLIP semantic embeddings and frequency patch features to capture both contextual cues and low-level visual artifacts. 7) C2P-CLIP (AAAI’2025) [51] proposed a CLIP-based AIGC detector, which introduces category common prompts into the text encoder to enhance category-related visual representations for detection. 8) FIRE (CVPR’2025) [38] proposed a frequency-guided reconstruction error method, which exploits the reconstruction discrepancy of mid-band frequency to detect diffusion-generated images.

#### Evaluation metrics.

We adopt 3 widely used evaluation metrics, detection accuracy(ACC), average precision(AP) and area under curve(AUC) in experiments, which are mainstream image forgery detection methods in previous research. All results are reported as the mean ± std deviation across 5 independent runs with distinct random seeds.

### Implementation details

All input images are uniformly resized to 512 × 512 pixels and normalized to the [0, 1] range. Images are then encoded into latent space with pretrained VAE. A probing perturbation is added on images in latent space, followed by diffusion reconstruction. The pre-/post-reconstruction error is used to construct detection features, which are subsequently fed into a classifier.

### Reconstruction configuration

We use Stable Diffusion v1.5, implemented under the Latent Diffusion Model (LDM) framework, with publicly available pretrained weights [26]. The diffusion model parameters are kept frozen, without any fine-tuning or retraining during all experiments.

In diffusion reconstruction stage, we employ the DDIM sampler with 25 denoising steps, 0.6 reconstruction strength, and a linear noise schedule. Real and diffusion-generated images are reconstructed with identical settings. We adopt unconditional reconstruction (empty prompt) and set the guidance scale to 1.0 to avoid introducing additional semantic priors from text conditioning.

### Probing perturbation

Probing perturbation is optimized in latent space, parameterized as directional vectors with an explicit 2-norm constraint (ϵ=30). Optimization is performed using Adam with a learning rate of 0.15 for 30 iterations.

In each iteration, the same probe is applied simultaneously to latent representations of both real and diffusion-generated images, and the reconstruction responses are computed. To obtain backpropagation gradients through the diffusion model, the reconstruction response is estimated via Monte Carlo sampling, with 8 samples per estimate. Real and diffusion-generated images share identical parameter configurations during perturbation addition, diffusion reconstruction, and discrepancy computation. 8 real–generated image pairs are randomly selected per class for perturbation learning, with a batch size of 16.

### Classifier

The reconstruction difference tensor is fed into a CNN-based binary classifier in experiments. The network input is a 4 × 64 × 64 latent tensor. Discriminative features are extracted through 4 cascaded convolutional modules, followed by adaptive global average pooling and a fully connected layer to output the binary prediction. The classifier architecture is kept identical across all experiments, and it is used solely to evaluate detection performance under different diffusion reconstruction difference settings.

### Hardware

All experiments are conducted with NVIDIA TITAN RTX (24GB VRAM). The deep learning framework is PyTorch 2.5.1 + cu120, and diffusion inference pipeline is implemented with the official Diffusers library. Random seeds are fixed at key steps to ensure reproducibility of the results.

### Generalization evaluation

#### Single-generator training evaluation.

**Fig 5:**
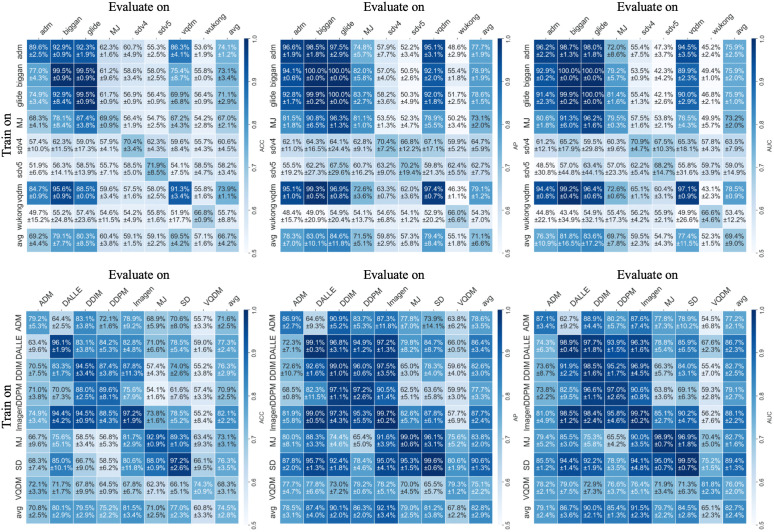
Comparison of classification performance across different datasets and diffusion generators. From left to right, the figure reports ACC, AP and AUC. The top row shows the cross-validation results on GenImage, while the bottom row shows the results on WildFake. Each subplot presents detection performance under different diffusion generators, where the x-axis denotes the test subsets (corresponding to different diffusion generators) and the y-axis indicates the source generator used for training.

We first evaluate the cross-generator generalization ability of our model on GenImage. Specifically, the model is trained on images from one diffusion generator, and tested on images from the remaining generators. Each experiment is repeated with 5 random seeds, and the results are averaged. The overall results are shown in Fig 5.

(1) Detecting images generated by seen generators is relatively easy. When the train and test sets come from the same generator, our method exhibits stable performance across all evaluation metrics. This indicates that the proposed detection framework can effectively capture reconstruction difference between real and diffusion-generated images under specific generator.
(2) Generalization varies substantially depending on the generator used for training. Among all training sources, the model trained on Imagen-generated images from WildFake demonstrates the strongest generalization ability, achieving relatively consistent detection performance across multiple unseen generators. Specifically, it reaches ACC/AP/AUC of 82.1 ± 2.2%, 87.7 ± 2.4%, and 88.1 ± 2.2, respectively.
(3) Overall performance is better when testing on WildFake dataset. This is mainly attributable to distributional differences between the 2 datasets. Images in WildFake are relatively constrained in terms of semantic consistency, and the distribution gap between real and diffusion-generated images is more pronounced. As a result, the detection task becomes inherently easier, leading to a relatively better overall performance.

#### Leave-one-model-out evaluation.

To evaluate whether ADRD can transfer to unseen generators, we adopt a leave-one-model-out protocol, where one generator is excluded from training and used only for testing. As shown in Table 1, ADRD achieves average ACC, AP, and AUC scores of 61.2%, 65.1%, and 65.9%, respectively. These results are lower than those obtained under the standard setting, which is expected because the detector must rely on reconstruction-discrepancy patterns learned from other generators rather than generator-specific cues. Still, the performance remains above chance in most cases, suggesting that ADRD captures features with certain cross-model transferability.

**Table 1:** Leave-one-model-out evaluation of ADRD across different diffusion generators.

Metric	ADM	BigGAN	Glide	Midjourney	SDV1.4	SDV1.5	VQDM	Wukong	Average
ACC	60.8 ± 10.5	83.8 ± 13.8	70.7 ± 16.5	59.7 ± 5.9	55.9 ± 5.3	52.6 ± 0.9	56.5 ± 4.3	49.9 ± 5.7	61.2 ± 7.9
AP	64.0 ± 12.5	89.5 ± 4.0	79.9 ± 16.2	60.1 ± 13.4	58.4 ± 4.9	58.3 ± 5.9	59.1 ± 15.5	51.6 ± 3.5	65.1 ± 9.5
AUC	63.9 ± 12.0	90.2 ± 5.8	77.5 ± 15.8	59.6 ± 13.3	62.7 ± 5.9	62.3 ± 7.0	57.5 ± 13.2	53.9 ± 4.7	65.9 ± 9.7

One generator is held out for testing while the remaining generators are used for training. Results are reported as mean ± standard deviation.

#### Mixed-training evaluation.

We further evaluate ADRD in a mixed-training setup, where all generators are combined during training and the trained model is evaluated separately on each generator. Table 2 summarizes the results. On average, ACC, AP, and AUC reach 68.9%, 74.9%, and 73.9%, corresponding to absolute improvements of 7.7, 9.8, and 8.0 percentage points over the leave-one-model-out baseline. This pattern suggests that exposing ADRD to more diverse generator distributions help it learn reconstruction-discrepancy cues that are less tied to a single source. Improvements are especially visible on ADM, Glide, Midjourney, SD v1.5, and VQDM. At the same time, SD v1.4 and Wukong show relatively limited gains, indicating that simply mixing more generators into the training pool is not sufficient to fully close the cross-model generalization gap.

**Table 2:** Mixed-training evaluation of ADRD across different diffusion generators.

Metric	ADM	BigGAN	Glide	Midjourney	SDV1.4	SDV1.5	VQDM	Wukong	Average
ACC	66.2 ± 1.4	77.8 ± 8.0	86.2 ± 5.2	72.0 ± 9.0	57.0 ± 8.7	64.5 ± 9.0	73.2 ± 5.0	54.5 ± 9.0	68.9 ± 6.9
AP	78.9 ± 5.8	93.7 ± 4.3	92.0 ± 3.3	75.3 ± 5.7	64.9 ± 5.5	63.9 ± 8.7	76.9 ± 4.5	53.8 ± 9.3	74.9 ± 5.9
AUC	77.3 ± 7.9	91.1 ± 7.5	92.4 ± 4.0	75.6 ± 9.0	65.6 ± 6.5	63.8 ± 10.2	71.6 ± 5.7	53.8 ± 11.5	73.9 ± 7.8

Samples from multiple generators are jointly used for training, and evaluation is performed separately on each generator. Results are reported as mean ± standard deviation.

### Compare with the state of the arts

We compare ADRD with different detection methods on GenImage benchmark, shown in Table 3. Early GAN-based detectors [47,48] work poorly on diffusion images, with both ACC and AP below 60%. While universal detectors [16,35,49] despite incorporating optimizations for diffusion models, their performance varies significantly across generators, typically achieving ACC/AP between 55% and 75%. Recent methods like AIDE [50], C2P-CLIP [51], and FIRE [38] further improve the overall performance, especially in terms of AP.

**Table 3:** Comprehensive comparisons between our ADRD and baselines.

Method	Testing diffusion generators								Total Avg
	ADM	BigGAN	Midjourney	SDV1.4	SDV1.5	Wukong	VQDM	Glide	
CNNSpot	59.4/70.7	49.7/50.0	52.7/57.1	51.4/57.4	52.5/58.9	50.1/53.6	52.2/59.1	52.4/63.3	52.5/57.7
Gram	57.7/56.8	53.8/54.0	60.1/58.3	64.0/61.9	66.0/63.6	63.5/61.0	55.2/52.9	55.6/54.0	57.1/58.2
UnivFD	68.3/**90.6**	50.7/64.6	49.3/48.8	61.6/67.4	51.60/66.6	54.7/79.2	**79.0/96.2**	57.7/84.6	57.7/74.7
LNP	70.5/77.0	**79.6**/92.3	54.1/58.4	60.8/68.2	63.4/72.3	61.0/68.8	57.3/58.5	67.0/69.5	64.2/70.7
DIRE	59.9/84.2	54.7/65.2	63.0/82.0	71.7/82.3	71.7/82.3	72.3/82.0	61.4/85.8	67.1/93.7	63.2/82.2
AIDE	56.0/81.5	62.4/92.3	68.7/**86.9**	57.4/**85.5**	55.1/84.1	58.4/86.2	59.0/89.9	54.0/78.2	58.9/**85.8**
C2P-CLIP	60.3/79.3	59.3/75.2	56.6/67.4	**77.5/**83.5	**76.9/85.0**	**79.4/92.1**	61.1/76.6	73.5/85.6	68.1/80.6
FIRE	**72.3**/87.4	74.7/**93.9**	63.4/70.6	62.5/71.9	68.9/77.0	61.6/67.0	64.6/74.5	75.0/89.0	67.9/78.9
ADRD (ours)	66.2 ± 1.4/ 78.9 ± 5.8	77.8 ± 8.0/ 93.7 ± 4.3	**72.0 ± 9.0/** 75.3 ± 5.7	57.0 ± 8.7/ 64.9 ± 5.5	64.5 ± 9.0/ 63.9 ± 8.7	54.5 ± 9.0/ 53.8 ± 9.3	73.2 ± 5.0/ 76.9 ± 4.5	**86.2 ± 5.2/** **92.0 ± 3.3**	**68.9 ± 6.9/** 74.9 ± 5.9

We download the pre-trained models officially provided by CNNSpot, Gram, UnivFD, LNP, DIRE, AIDE, C2P-CLIP and FIRE for evaluation. All data represent averages obtained from testing on various subsets of GenImage. For ADRD, both ACC(%) and AP(%) are reported as mean ± std in Table (ACC/AP).

Under the same evaluation protocol, ADRD achieves an overall ACC/AP of 68.9 ± 6.9% / 74.9 ± 5.9%. Compared with existing methods, ADRD obtains competitive average accuracy and performs favorably on several generators. However, ADRD does not consistently outperform all competing methods. In particular, its average AP remains lower than several recent detectors. This indicates that although the proposed reconstruction-response signal is effective, it does not fully replace the stronger discriminative cues captured by recent detection models.

We attribute this gap to the inherent variability of reconstruction sensitivity across generators. For SD v1.4, SD v1.5, and Wukong, the probe-induced discrepancy separates real and generated samples less clearly, leading to weaker AP. Overall, ADRD offers a complementary perspective: instead of relying on passive reconstruction errors or visual features, it actively probes how real and generated images respond differently to optimized latent perturbations.

### Reconstruction discrepancy analysis

To evaluate the effect of the learned probe, we compare the latent-space reconstruction discrepancy under 3 settings: without probe, random probe, and optimized probe. The discrepancy is measured by the $L_{\text{2}}$ distance between the perturbed latent representation and its reconstruction.

**Fig 6:**
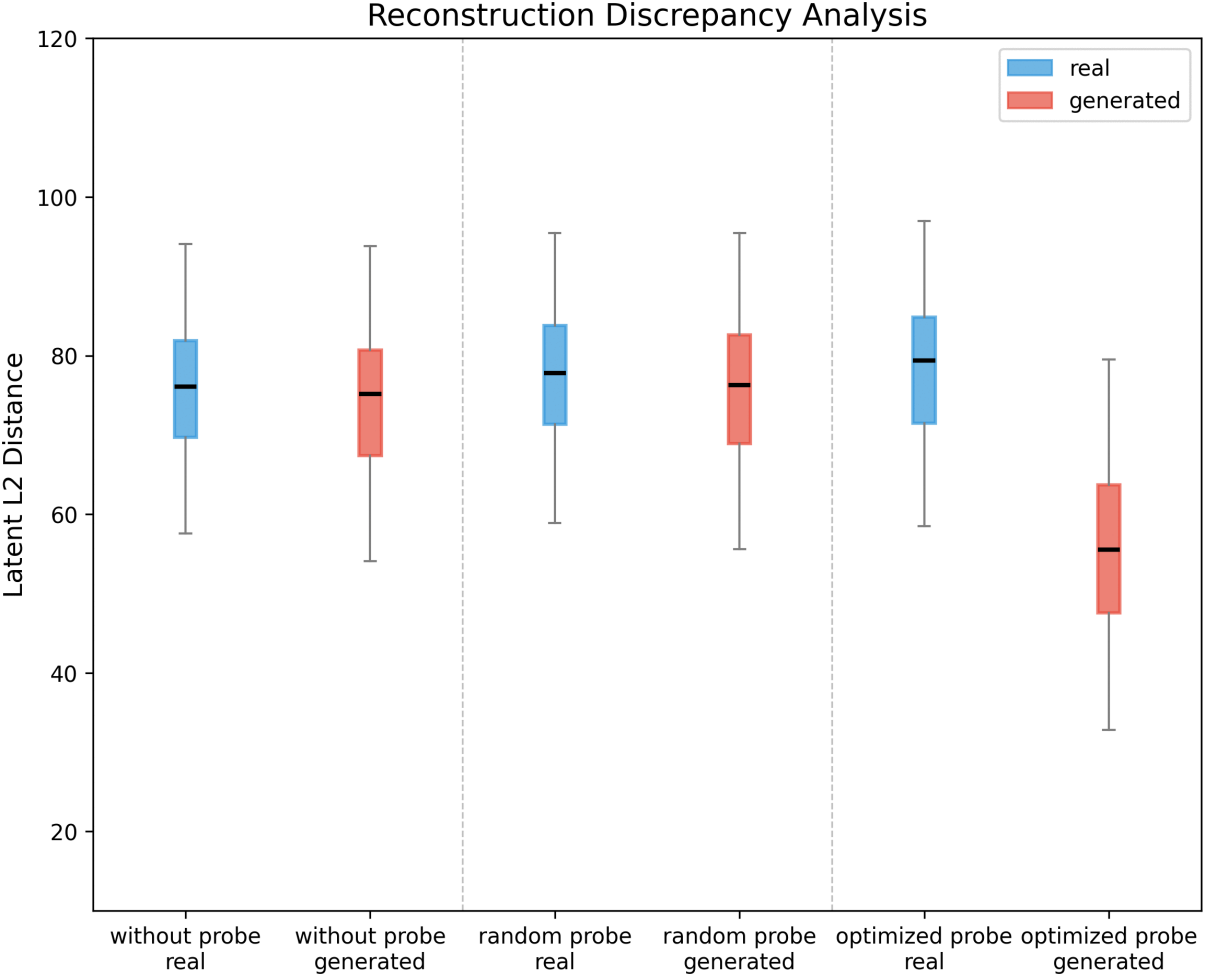
Reconstruction discrepancy distributions under different probing settings. Box plots compare discrepancies between real and diffusion-generated images for without probe, random probe, and optimized probe conditions. The optimized probe yields the clearest separation.

As shown in Fig 6, real and generated samples have highly overlapping discrepancy distributions without probe applied. The random probe leads to only a small shift in the distributions, and the 2 classes remain difficult to separate. By contrast, the optimized probe increases the discrepancy of real images more substantially, while generated images remain concentrated in a lower discrepancy range.

This result indicates that the optimized probe is not equivalent to injecting random noise. Instead, it exposes differences in the reconstruction responses of real and diffusion-generated images, making the latent space reconstruction discrepancy more useful for detection.

## Ablation study

### Probing perturbation

To assess the contribution of probing perturbation in latent space to detection performance, we compared 3 settings under the same training/testing protocol: Without probe, Random probe, and Optimized probe (learned through our optimization strategy). ACC/AP are reported separately for 8 diffusion generators (ADM, BigGAN, Glide, Midjourney, SD v1.4, SD v1.5, VQDM, Wukong). All results represent the mean ± std deviation from 5 independent trials, as shown in Table 4.

**Table 4:** Detection performance under different probing perturbation settings across image generators.

Method		Testing diffusion generators								
		ADM	BigGAN	Glide	Midjourney	SDV1.4	SDV1.5	VQDM	Wukong	Total Avg
Without probe	ACC	62.3 ± 3.9	71.0 ± 4.9	74.8 ± 7.3	57.2 ± 2.6	47.5 ± 1.0	50.5 ± 2.6	63.2 ± 2.2	44.2 ± 1.4	58.8 ± 3.3
	AP	71.7 ± 3.6	76.1 ± 3.2	82.3 ± 7.9	66.1 ± 2.6	51.8 ± 1.6	53.4 ± 4.8	72.9 ± 2.4	49.2 ± 3.3	65.4 ± 3.7
Random probe	ACC	65.7 ± 3.0	72.5 ± 4.2	76.3 ± 5.2	59.5 ± 1.4	49.2 ± 1.4	52.0 ± 1.6	65.3 ± 2.0	46.4 ± 1.3	60.9 ± 2.5
	AP	72.3 ± 1.8	74.3 ± 2.9	80.0 ± 6.1	66.3 ± 1.8	50.9 ± 2.1	51.8 ± 2.6	71.1 ± 2.5	49.9 ± 1.4	64.6 ± 2.7
Optimized probe	ACC	**69.2 ± 4.4**	**79.1 ± 7.7**	**80.3 ± 8.5**	**60.4 ± 3.8**	**59.1 ± 1.5**	**59.1 ± 2.2**	**69.5 ± 4.2**	**57.1 ± 1.6**	**66.7 ± 4.2**
	AP	**78.3 ± 7.0**	**83.0 ± 10.1**	**84.6 ± 11.8**	**71.5 ± 5.1**	**59.8 ± 2.9**	**57.3 ± 5.8**	**79.4 ± 8.4**	**55.1 ± 1.8**	**71.1 ± 6.6**

ACC(%) and AP(%) are reported as mean ± std above.

The results demonstrate that introducing probing perturbation enhances detection performance to some extent, with the optimized probe outperforming the random probe. Compared with the Random probe, the optimized probe improves the overall average ACC/AP from 60.9 ± 2.5% / 64.6 ± 2.7% (ACC/AP) to 66.7 ± 4.2% / 71.1 ± 6.6%, corresponding to absolute improvements of +5.8 / + 6.5 percentage points. This phenomenon suggests that perturbation optimization targeting the “reconstruction discrepancy” objective can yield more discriminative reconstruction responses, rather than merely introducing additional noise.

### Image pairs in optimizing probe

**Fig 7:**
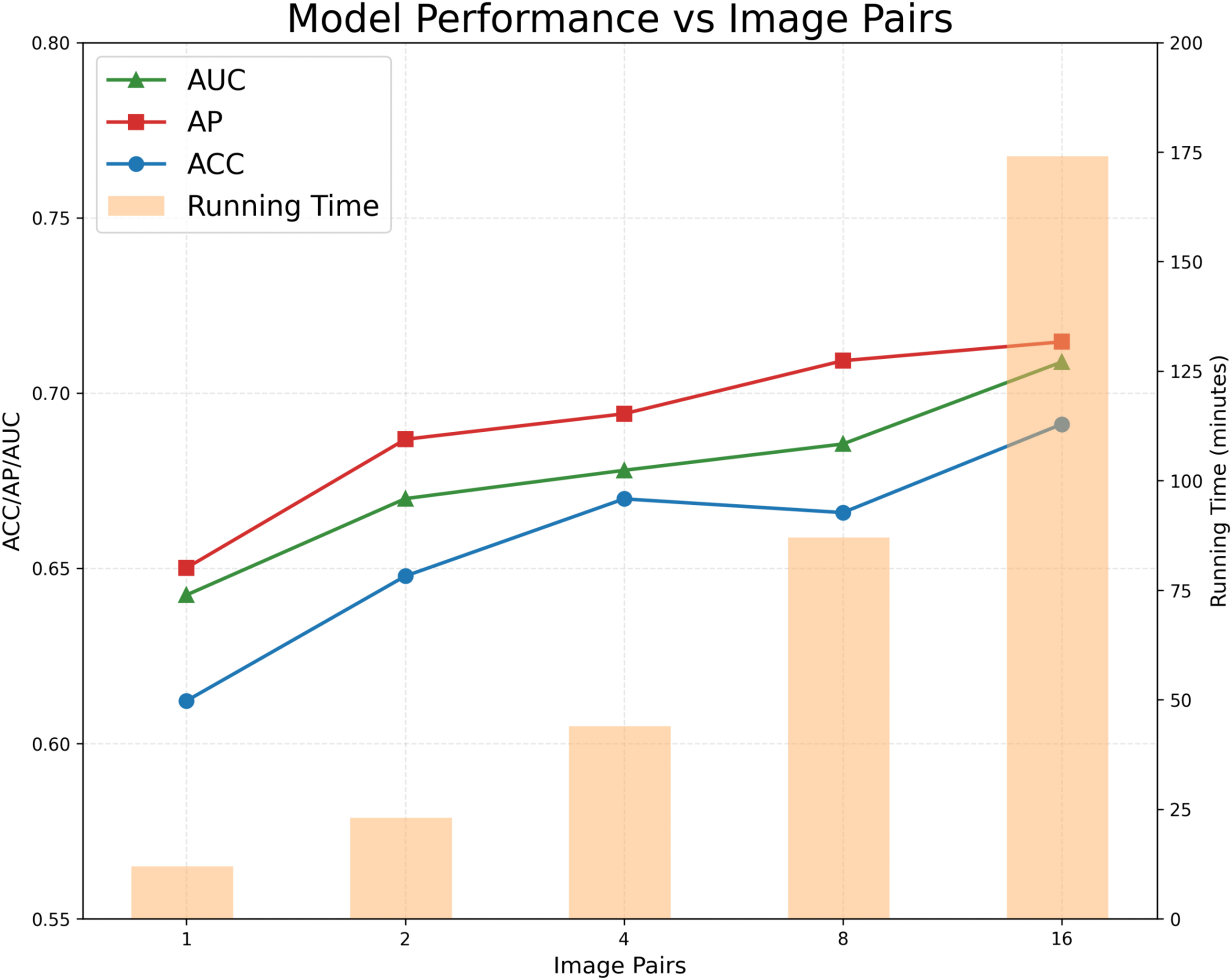
Impact of the number of image pairs on detection performance and computational cost during the perturbation optimization phase.

To enhance the cross-generator applicability of probing perturbation, we further investigate the impact of different numbers of “real – generated image pairs” during the perturbation optimization phase on final detection performance and computational cost, as shown in Fig 7. The results indicate that as the number of image pairs increased, the detection performance (ACC/AP/AUC) generally shows an upward trend, suggesting that more pairs provide a more stable optimization signal. However, concurrently, the runtime for perturbation optimization also significantly increases. Based on the magnitude of detection performance improvement balanced against the runtime overhead, we select 8 image pairs as the default setting for subsequent experiments. This setting achieves performance close to that obtained with larger sets of image pairs while preventing a significant runtime increase, thus striking a reasonable trade-off between effectiveness and efficiency.

The figure compares the trends observed when using different numbers of “real image–diffusion-generated image pairs” for learning probing perturbation. As the number of image pairs increases, metrics (ACC/AP/AUC) improve overall, but the runtime also increases accordingly, demonstrating a trade-off between performance and computational cost.

### Diffusion reconstruction

To further analyze the role of diffusion reconstruction in ADRD, we conducted an ablation study on **strength** parameter. This parameter controls the level of noise injected into the input before reconstruction, thereby establishing a continuous trade-off between “preserving input structural information” and “relying on the generative prior of diffusion model.”

**Fig 8:**
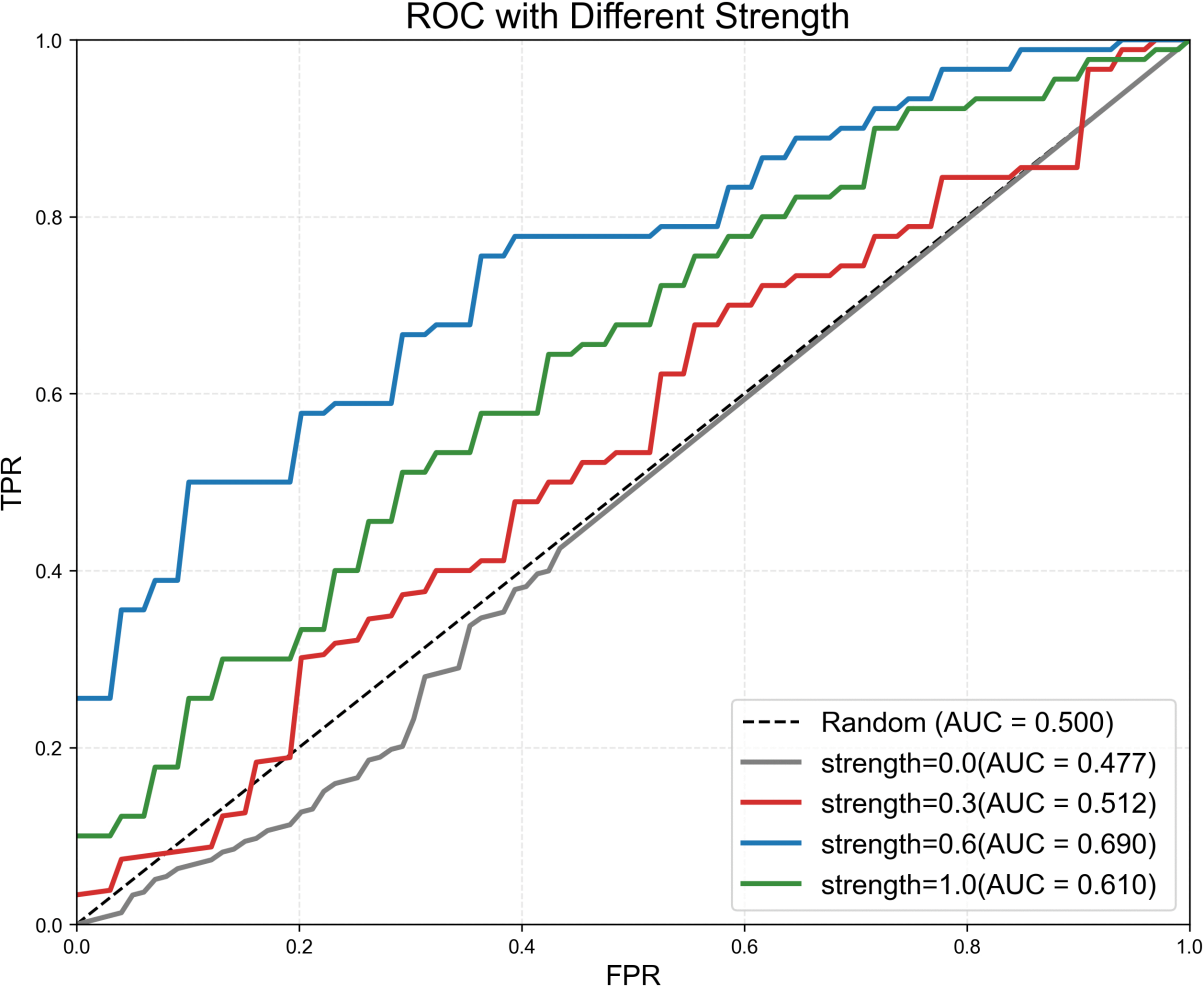
Comparison of ROC curves under different diffusion reconstruction strengths (on GenImage). The x-axis represents FPR, and the y-axis represents TPR. Detection performance exhibits significant variation with changes in strength: the legend provides the corresponding AUC values for each setting.

When strength = 0, the reconstruction effectively degenerates into an approximate identity mapping. Consequently, the resulting reconstruction error is near-zero, failing to elicit sufficient discriminative differences in reconstruction stability. This leads to detection performance close to random guessing (AUC = 0.477). Conversely, when strength = 1.0, the input is completely noised. The reconstruction is then primarily governed by the generative prior of diffusion model, significantly weakening the individual characteristics of the input image. This causes the reconstruction of 2 image classes to become more homogeneous, partially obscuring discriminative signals, and results decrease compared to intermediate strength values (AUC = 0.610). As illustrated in Fig 8, reconstruction with an intermediate strength value (strength = 0.6) achieved the best performance (AUC = 0.690). This indicates that within this range, the reconstruction process retains sufficient input structure while simultaneously introducing adequate noise and reverse diffusion iterations, thereby generating stronger discriminative signals for detection. Therefore, a strength value of 0.6 was adopted as the default setting for subsequent experiments.

### Impact of different classifiers

In our experiments, we compared 4 different classification methods: (1) Support Vector Machine (SVM) as a traditional machine learning baseline; (2) SimpleCNN, a shallow convolutional neural network; (3) BetterCNN, a convolutional neural network with deeper feature extraction capabilities; (4) AttentionCNN, a convolutional neural network incorporating an attention mechanism.

The performance comparison for these classifiers across 3 metrics (ACC/AP/AUC) is presented in Table 5. The results indicate notable performance differences among classifiers. SVM demonstrates relatively lower overall performance, suggesting that the reconstruction difference features are not entirely linearly separable. In contrast, SimpleCNN, by introducing a convolutional structure, effectively leverages local spatial information within the reconstruction difference tensor, achieving significant improvements across all metrics. BetterCNN achieves the optimal results in ACC/AP/AUC, indicating that deeper feature extraction facilitates the capture of more subtle variations in the reconstruction error within latent space. Although AttentionCNN introduces an explicit attention mechanism, its performance improvement was limited; this is likely due to the low spatial resolution of latent space difference tensor. Overall, the experimental results demonstrate that BetterCNN achieves the best balance between performance and model complexity.

**Table 5:** Performance comparison of different classifiers on GenImage.

Method		Testing diffusion generators								
		ADM	BigGAN	Glide	Midjourney	SDV1.4	SDV1.5	VQDM	Wukong	Total Avg
SVM	ACC	62.3 ± 0.0	68.2 ± 0.0	75.2 ± 0.0	**61.1 ± 0.0**	47.5 ± 0.0	44.9 ± 0.0	57.0 ± 0.0	43.0 ± 0.0	57.4 ± 0.0
	AP	54.8 ± 6.5	61.1 ± 9.8	64.6 ± 12.6	54.2 ± 6.3	52.3 ± 0.9	51.1 ± 1.5	58.1 ± 4.4	53.2 ± 4.1	56.2 ± 5.7
	AUC	52.2 ± 6.9	57.5 ± 12.2	57.3 ± 15.3	50.6 ± 7.5	51.0 ± 0.9	49.6 ± 1.8	53.4 ± 4.3	**52.7 ± 4.3**	53.0 ± 6.6
Simple CNN	ACC	65.0 ± 2.7	76.1 ± 4.6	77.7 ± 5.3	59.3 ± 2.2	50.5 ± 1.3	52.7 ± 2.0	65.3 ± 1.7	47.7 ± 3.0	61.8 ± 2.9
	AP	72.5 ± 2.7	77.6 ± 3.4	82.0 ± 3.2	66.9 ± 2.2	52.9 ± 3.3	55.9 ± 2.5	73.1 ± 2.7	49.0 ± 2.6	66.2 ± 2.8
	AUC	71.9 ± 3.5	78.6 ± 7.7	80.7 ± 6.1	67.1 ± 3.1	53.5 ± 2.5	53.7 ± 2.5	71.6 ± 3.4	45.3 ± 2.6	65.3 ± 3.9
Better CNN	ACC	**69.2 ± 4.4**	**79.1 ± 7.7**	**80.3 ± 8.5**	60.4 ± 3.8	**59.1 ± 1.5**	**59.1 ± 2.2**	**69.5 ± 4.2**	**57.1 ± 1.6**	**66.7 ± 4.2**
	AP	**78.3 ± 7.0**	**83.0 ± 10.1**	**84.6 ± 11.8**	**71.5 ± 5.1**	**59.8 ± 2.9**	**57.3 ± 5.8**	**79.4 ± 8.4**	**55.1 ± 1.8**	**71.1 ± 6.6**
	AUC	**76.3 ± 10.9**	**81.8 ± 16.5**	**83.6 ± 17.2**	**69.7 ± 7.8**	**59.5 ± 2.3**	**54.7 ± 4.3**	**77.4 ± 11.5**	52.3 ± 1.5	**69.4 ± 9.0**
Attention CNN	ACC	64.8 ± 4.1	74.8 ± 5.6	77.2 ± 8.0	58.2 ± 3.3	53.3 ± 2.6	53.1 ± 1.2	64.3 ± 2.1	48.2 ± 2.0	61.7 ± 3.6
	AP	72.6 ± 6.9	77.4 ± 8.7	81.8 ± 10.6	65.2 ± 4.4	55.9 ± 2.6	54.3 ± 6.0	72.4 ± 2.9	49.7 ± 4.2	66.2 ± 5.8
	AUC	71.0 ± 7.1	76.8 ± 12.0	78.9 ± 15.7	66.3 ± 5.4	57.8 ± 1.8	51.8 ± 5.0	70.5 ± 4.4	47.5 ± 3.4	65.1 ± 6.8

ACC(%), AP(%) and AUC(%) are reported as mean ± std above.

### Robustness to common propagation degradations

**Fig 9:**
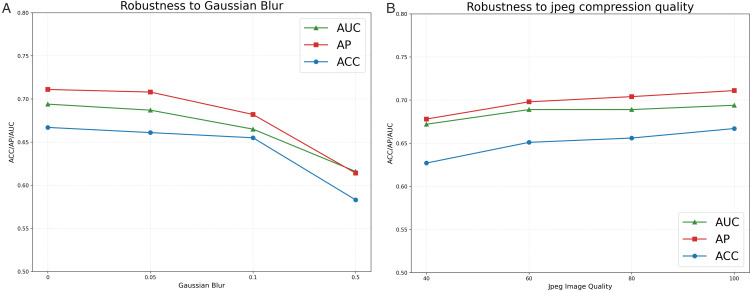
Robustness to common propagation degradations. (A) Robustness against different Gaussian blur. (B) Robustness against different JPEG compression. (A) It indicates robustness against different levels of Gaussian blur, where larger σ signifies higher blur. (B) Robustness under different JPEG quality levels, where a higher quality value indicates lower compression. ACC(%), AP(%) and AUC(%) are reported above.

Beyond generalization capability for unseen diffusion-generated images, robustness against unseen interference constitutes another critical evaluation metric. In actual applications, images transmitted through network platforms are inevitably subjected to noise and compression. Therefore, we evaluated the robustness of ADRD under 2 interference types: Gaussian blur and JPEG compression. The degradations are applied at 4 levels for Gaussian blur σ=0, 0.05, 0.1, 0.5 in pixel space and 4 levels for JPEG compression quality=40, 60, 80, 100. The test results for different diffusion-generated images are shown in Fig 9. We observe that the detection performance (ACC/AP/AUC) exhibited a decline under increasing Gaussian blur intensity. This degradation became more pronounced when σ exceeded 0.1. In contrast, across the tested range of JPEG compression levels, the 3 metrics (ACC/AP/AUC) demonstrate minimal variation, indicating that ADRD possesses good stability against common compression distortions.

### Computational complexity analysis

We report the computational costs associated with our proposed method. This includes the reconstruction time per image, classifier inference time per image, and total processing time. We further convert the total time into GPU hours required to process 8,000 test images. These measurements exclude the computational time required for probing perturbation optimization. All measurements were conducted on a single NVIDIA TITAN RTX GPU, with a batch size of 4, an input resolution of 256 × 256, and a total of 8,000 images.

As illustrated in Table 6, the primary computational overhead in ADRD stems from the diffusion reconstruction. The additional overhead introduced by subsequent difference calculation and CNN-based classifier inference is negligible. Under the default DDIM reconstruction settings, ADRD requires approximately 0.94 GPU hours to process 8,000 images, thus making it suitable for offline analysis. Compared to lightweight detectors that do not rely on reconstruction, ADRD exhibits higher latency per image. Its applicability in scenarios with stringent real-time requirements remains constrained by computational cost. Future work will explore more efficient reconstruction strategies and acceleration techniques to further reduce GPU resource consumption.

**Table 6:** Computational complexity comparison of different methods.

Method	ACC/AP	Reconstruct time (ms)	Inference time (ms)	Total time (ms)	GPU hours/8k images
CNNSpot	52.5/57.7	--	17	17	0.038 hours
DIRE	63.2/82.2	4950	17	4967	11.04 hours
ADRD (Ours)	66.7/71.1	420	2	422	0.94 hours

CNNSpot and DIRE were evaluated with their officially provided pre-trained models. ADRD was evaluated with the model implemented in this study. The table reports the reconstruction time per image, inference time per image, and total processing time per image. It also provides the GPU hours required to process 8,000 images.

## Conclusion

This paper proposes ADRD for the detection of diffusion-generated images. The method is based on a key observation: the inversion-reconstruction process of diffusion models, constrained by the model’s prior, can be viewed as a “distribution alignment” mapping. Images originating from different underlying distributions exhibit varying sensitivities under this mapping. To amplify this difference, we introduce an active probing perturbation in latent space. The detection signal is then constructed from the reconstruction response differences under this perturbed condition. Finally, a lightweight classifier is employed for discrimination.

Experimental results, validated across multiple diffusion models, cross-dataset settings, and different reconstruction configurations, support our hypothesis: ADRD stably produces discriminative detection features. It demonstrates reasonable generalization capability, robustness, and acceptable computational overhead. Overall, ADRD provides a generalizable “probe–response” paradigm for leveraging diffusion reconstruction sensitivity in detection, establishing it as an effective framework for detecting diffusion-generated image forgeries.

Despite ADRD’s stable performance across diverse settings, several limitations remain. Firstly, there is still room for improvement in detection performance on Stable Diffusion series models; the overall metrics may still fall short of requirements for practical deployment. Secondly, the optimization objective for the probing perturbation could be further refined, potentially incorporating joint optimization using distribution distance metrics like the Fréchet Inception Distance (FID) or consistency constraints, indicating potential for enhancing the probing perturbation strategy.

## Supporting information

S1 Table 95% confidence intervals (CIs) corresponding to the ablation study in Table 4. The CIs of ACC (%) and AP (%) are computed across 5 independent random seeds using the Student’s t-distribution for each probing perturbation setting and diffusion generator, as well as for the overall average performance.
(DOCX)

S2 Table 95% confidence intervals (CIs) corresponding to the ablation study in Table 5. The CIs of ACC (%), AP (%) and AUC(%) are computed across 5 independent random seeds using the Student’s t-distribution for each classification methods and diffusion generator, as well as for the overall average performance.
(DOCX)
