# Supplementary material for: ADRD: Detecting diffusion-generated images via adversarial perturbation induced reconstruction discrepancy
Source: PLoS One. 2026 Jul 14;21(7):e0350655. doi: 10.1371/journal.pone.0350655 (PMC13367716; doi:10.1371/journal.pone.0350655)
Supplement: S1 Table — The CIs of ACC (%) and AP (%) are computed across 5 independent random seeds using the Student’s t-distribution for each probing perturbation setting and diffusion generator, as well as for the overall average performance. (DOCX) [file pone.0350655.s001.docx]

**S1 Table: 95% confidence intervals (CIs) corresponding to the ablation study in Table 2.**

The CIs of ACC (%) and AP (%) are computed across 5 independent random seeds using the Student’s t-distribution for each probing perturbation setting and diffusion generator, as well as for the overall average performance.

| Method | | Testing diffusion generators | | | | | | | | |
| --- | --- | --- | --- | --- | --- | --- | --- | --- | --- | --- |
|  |  | ADM | Biggan | Glide | Midjourney | SDV1_4 | SDV1_5 | VQDM | wukong | Total Avg |
| Without  probe | ACC | (52.5–76.2) | (59.7–86.3) | (58.0–95.6) | (48.3–70.2) | (43.3–55.8) | (42.9–62.0) | (55.4–75.0) | (40.5–52.0) | (49.4–72.3) |
|  | AP | (60.8–82.5) | (65.6–86.6) | (58.6–100) | (56.0–76.2) | (45.7–57.9) | (37.3–69.6) | (65.2–80.5) | (40.2–58.2) | (52.6–78.3) |
| Random  probe | ACC | (55.0–76.4) | (60.1–85.0) | (61.6–91.0) | (53.6–65.4) | (42.1–56.3) | (45.0–58.9) | (58.4–72.2) | (39.0–53.9) | (51.4–70.4) |
|  | AP | (64.0–80.6) | (64.7–83.9) | (64.0–96.0) | (60.4–72.3) | (43.9–58.0) | (42.5–61.2) | (63.1–79.2) | (44.9–55.0) | (55.4–73.8) |
| Optimized  probe | ACC | (57.0–75.1) | (63.2–85.3) | (64.7–94.5) | (49.1–68.6) | (43.5–57.0) | (42.5–63.1) | (57.1–73.2) | (39.2–55.9) | (51.8–71.9) |
|  | AP | (60.5–85.7) | (63.6–92.2) | (62.3–100) | (56.7–76.0) | (42.6–61.7) | (40.3–66.3) | (64.3–81.1) | (41.2–59.1) | (53.3–79.0) |
