# Supplementary material for: ADRD: Detecting diffusion-generated images via adversarial perturbation induced reconstruction discrepancy
Source: PLoS One. 2026 Jul 14;21(7):e0350655. doi: 10.1371/journal.pone.0350655 (PMC13367716; doi:10.1371/journal.pone.0350655)
Supplement: S2 Table — The CIs of ACC (%), AP (%) and AUC(%) are computed across 5 independent random seeds using the Student’s t-distribution for each classification methods and diffusion generator, as well as for the overall average performance. (DOCX) [file pone.0350655.s002.docx]

**S2 Table: 95% confidence intervals (CIs) corresponding to the ablation study in Table 3.**

The CIs of ACC (%), AP (%) and AUC(%) are computed across 5 independent random seeds using the Student’s t-distribution for each classification methods and diffusion generator, as well as for the overall average performance.

| Method | | Testing diffusion generators | | | | | | | | |
| --- | --- | --- | --- | --- | --- | --- | --- | --- | --- | --- |
|  |  | ADM | Biggan | Glide | Midjourney | SDV1_4 | SDV1_5 | VQDM | wukong | Total Avg |
| SVM | ACC | (62.3–62.3) | (68.2–68.2) | (75.2–75.2) | (61.1–  61.1) | (47.5–47.5) | (44.9–44.9) | (57.0–57.0) | (43.0–43.0) | (57.4–57.4) |
|  | AP | (41.1–68.6) | (39.0–83.3) | (36.2–92.9) | (39.9–  68.5) | (50.3–54.3) | (47.7–54.5) | (48.4–67.8) | (44.5–61.9) | (40.9–71.5) |
|  | AUC | (37.6–66.8) | (30.1–84.8) | (22.9–91.6) | (34.0–  67.2) | (48.9–53.1) | (45.6–53.6) | (43.7–63.1) | (43.3–62.1) | (34.9–71.1) |
| SimpleCNN | ACC | (54.0–76.0) | (61.8–90.4) | (61.7–93.8) | (51.2–  67.4) | (43.7–57.4) | (45.1–60.3) | (55.0–75.6) | (39.3–56.1) | (51.0–72.6) |
|  | AP | (62.8–82.3) | (66.7–88.6) | (72.5–91.6) | (58.5–  75.2) | (42.2–63.6) | (46.9–64.9) | (62.4–83.9) | (40.4–57.5) | (56.5–76.0) |
|  | AUC | (59.6–84.3) | (58.1–99.1) | (64.5–97.0) | (56.0–  78.1) | (43.1–63.9) | (43.3–64.2) | (58.3–84.9) | (33.6–56.9) | (51.7–78.9) |
| BetterCNN | ACC | (49.2–78.8) | (57.7–92.3) | (56.7–99.1) | (45.4–  69.8) | (47.3–57.2) | (43.9–60.6) | (57.3–73.5) | (40.1–55.9) | (48.6–74.5) |
|  | AP | (53.9–88.5) | (60.9–90.8) | (64.5–96.6) | (52.6–  77.7) | (49.2–58.6) | (44.6–65.1) | (56.8–84.8) | (37.9–64.3) | (52.0–78.8) |
|  | AUC | (46.2–92.4) | (51.6–  100) | (52.4–100) | (51.8–  81.0) | (46.6–63.7) | (38.9–65.4) | (51.3–87.3) | (30.0–67.1) | (45.2–84.1) |
| AttentionCNN | ACC | (51.8–77.9) | (59.9–89.7) | (54.7–99.7) | (47.7–  68.7) | (44.6–62.1) | (46.8–59.4) | (55.0–73.6) | (40.2–56.1) | (49.1–74.3) |
|  | AP | (54.9–90.4) | (55.3–99.5) | (54.8–100) | (50.6–  79.9) | (48.1–63.6) | (37.8–70.8) | (62.2–82.6) | (37.1–62.3) | (49.0–83.3) |
|  | AUC | (51.3–90.8) | (47.5–  100) | (41.1–100) | (48.5–  84.2) | (49.7–66.0) | (36.7–66.8) | (57.7–83.3) | (33.7–61.3) | (43.8–86.4) |
